# Prostaglandin E2 in Brain Inflammation: Roles and Dynamics

**DOI:** 10.3390/brainsci16101118

**Published:** 2026-10-16

**Authors:** Takeo Nakanishi, Mamoru Fukuchi

**Affiliations:** 1 Laboratory of Biopharmaceutics, Faculty of Pharmacy, Takasaki University of Health and Welfare, Takasaki 370-0033, Gunma, Japan; 2 Laboratory of Molecular Neuroscience, Faculty of Pharmacy, Takasaki University of Health and Welfare, Takasaki 370-0033, Gunma, Japan; fukuchi@takasaki-u.ac.jp

**Keywords:** prostaglandin E_2_, EP receptor, neuroinflammation, neurological disorder, transporter, blood–brain barrier, blood–cerebrospinal fluid barrier

## Abstract

**Highlights:**

**What are the main findings?**

- PGE_2_/EP signaling regulates diverse and complex pathways in neurons and glial cells, influencing acute and chronic inflammation.
- Membrane transporters control local and brain PGE_2_ concentrations.

**What are the implications of the main findings?**

- Targeting PGE_2_/EP signaling offers promising therapeutic strategies for neuroinflammatory diseases.
- Unraveling the roles of PGE_2_ membrane transporters is vital to understanding the pathophysiological significance of PGE_2_ in the CNS.

**Abstract:**

**Background/Objectives**: Prostaglandin E2 (PGE_2_) is a known inflammatory mediator. It is produced by cyclooxygenase (COX)-1/2 during inflammation via four distinct G protein-coupled receptors (EP1–EP4). PGE_2_ is involved in the development of brain diseases induced by acute and chronic stresses, although the mechanisms regulating PGE_2_ concentration remain unclear. This review summarizes the mechanisms in relation to the activity of PGE_2_, focusing on membrane transporters in neuroinflammation and central nervous system (CNS) disorders. **Methodology**: A literature search was conducted using the PubMed database to evaluate the roles and dynamics of PGE_2_ in the central nervous system (CNS) under acute and chronic stress conditions. **Results**: PGE_2_ produced during inflammation acts via four receptors (EP1–EP4). These receptors mediate diverse and complex pathways in neurons and glial cells (microglia and astrocytes), influencing pathologies involving acute and chronic inflammation such as cerebral ischemia, Alzheimer’s disease, Parkinson’s disease, and neuropathic pain. EP1, EP2, and EP3 generally promote neurotoxicity, inflammation, and blood–brain barrier disruption, whereas EP4 predominantly exerts anti-inflammatory and neuroprotective effects. Furthermore, membrane transporters, particularly SLCO2A1 (also known as PGT) and MRP4 (encoded by ABCC4), regulate local PGE_2_ concentrations and facilitate its clearance across the blood–brain and blood–cerebrospinal fluid barriers. Understanding these transporters and receptors provides insights into controlling PGE_2_ dynamics in CNS diseases. **Conclusions**: Growing evidence indicates that membrane transporters play critical roles in regulating these levels in the brain, and elucidating the expression profiles of functional PG transporters may offer novel therapeutic strategies for refractory inflammation-based CNS diseases.

## 1. Introduction

Neuroinflammation is a critical driver and hallmark of numerous central nervous system (CNS) pathologies, ranging from acute conditions, such as infection-induced encephalitis, to chronic neurological disorders, including Alzheimer’s disease (AD), Parkinson’s disease (PD), and major depressive disorder. While acute neuroinflammatory responses aim to protect and repair injured neural tissues, sustained or dysregulated inflammation promotes the activation of glial cells, such as microglia and astrocytes, leading to breakdown of the blood–brain barrier (BBB) and progressive neuronal damage.

In the progression of these inflammatory cascades, prostaglandin E_2_ (PGE_2_) acts as a pivotal inflammatory mediator in the brain, playing essential roles through its cognate prostanoid E receptors (EP1–EP4) [1,2]. PGE_2_ is synthesized by virtually all neural and immune cell types in the brain, and is a major enzymatic product of cyclooxygenase-2 (COX-2) induced by inflammatory stimuli. Once produced and released, PGE_2_ acts via cell-surface EP receptors to mediate diverse signaling pathways—crucial for both maintaining brain homeostasis and modulating neuroinflammatory cascades—thereby exacerbating brain pathologies.

Beyond classical receptor-mediated signaling, emerging evidence reveals that membrane transporters governing PGE_2_ disposition are key determinants of its spatial and temporal biological functions as an autacoid [3,4].

This review summarizes the fundamental roles of PGE_2_ and its receptors in acute and chronic neuroinflammation, with a particular focus on how it controls the crosstalk between microglia and astrocytes to regulate the progression of CNS diseases. Furthermore, we discuss current insights into how membrane transporters regulate PGE_2_ dynamics in the inflamed brain, aiming to clarify their pathophysiological significance in neurological disorders.

## 2. Methodology

### 2.1. Study Design and Search Strategy

This review was conducted to evaluate the roles and dynamics of PGE_2_ in the CNS under acute and chronic stress conditions. A literature search was conducted using the PubMed database to identify relevant studies published from 1973 to 2026. The search strategy incorporated combinations of Medical Subject Headings (MeSH) terms and free-text keywords related to PGE_2_, stress, and specific neurological disorders. The exact search string was constructed by combining four core categories of keywords using Boolean operators. We targeted PGE_2_ within the context of acute or chronic stress and the CNS. This core context was then intersected with specific neurological pathologies, including brain ischemia, cerebral infarction, Alzheimer’s disease, Parkinson’s disease, and neuropathic pain. To further refine the roles and dynamics of PGE_2_, we integrated receptors and signaling molecules (EP1, EP2, EP3, and EP4) alongside membrane transporters (prostaglandin transporter, MRP4, and SLCO2A1).

### 2.2. Eligibility Criteria and Study Selection

To be included in the review, studies had to meet the following criteria: (1) peer-reviewed original research articles, (2) experimental studies focused on the roles and dynamics of PGE_2_, and (3) publications in English. Conversely, conference abstracts, commentaries, and case reports were excluded as much as possible. Full-text articles of potentially eligible records were retrieved and evaluated against predefined criteria. Any discrepancies during the screening process were resolved through discussion and consensus. Given the heterogeneity of the experimental designs and pathological models, a qualitative narrative synthesis was performed to integrate and present the findings.

## 3. PGE_2_ and Its Receptor Subtypes

PGE_2_ acts through four distinct G protein-coupled receptors (GPCR)—EP1, EP2, EP3, and EP4—which exhibit diverse signaling pathways, expression patterns, and physiological functions. The EP1 couples to G_q_ to increase intracellular Ca^2+^ and regulates pain transmission and is expressed in the kidney and CNS, while EP2 (G_s_-coupled) controls reproductive processes and vasodilation, with high expression in the uterus and vascular system. EP3 primarily mediates G_i_-linked inhibition of cAMP, which is crucial for fever and uterine contraction, and is widely expressed; conversely, EP4 (G_s_-coupled) activates PI3K/Akt signaling, which is essential for bone remodeling and immune responses in lymphoid tissues and the gut. EP2 and EP4 elevate intracellular levels of cAMP through G_s_, whereas EP3 decreases the level via G_i_ activation. In the CNS, EP1 and EP3 are expressed at relatively high levels in neurons [5,6], whereas EP4 expression is observed in certain neuronal nuclei [7]. In glial cells, EP4 and to a lesser extent EP2 are predominantly expressed in microglial cells, whereas all EP1–4 receptors are expressed at similar levels in astrocytes, suggesting key roles in microglial EP2 and EP4 in regulating inflammation and immune responses in the brain [8]. Thus, these receptors play a crucial role in maintaining the parenchymal environment and mediating responses to brain inflammation.

## 4. Prostaglandins and Their Membrane Transporters

Prostaglandins (PGs) are a subclass of eicosanoids and are found in nearly all tissues in the body. PGs are produced by every cell and act as local hormones to regulate various physiological functions necessary for survival, including blood pressure, digestive function, bronchoconstriction, and uterine contractions [9]. Physiologically active PGs include PGE_2_, prostacyclin (PGI_2_), PGD_2_, and PGF_2α_. PGE_2_, in particular, is a well-known lipid mediator of inflammation in various diseases, as it is the major product of COX-2 and PGES upregulated during inflammation. PGE_2_ exerts diverse and complex effects via the four distinct EP subtypes described above. PGE_2_-mediated signal is terminated by metabolic inactivation of 15-hydroxyprostaglandin dehydrogenase (15-PGDH/HPGD) into 15-ketoPGE_2_. The enzyme is primarily localized in the cytosol of cells; therefore, PGE_2_ cannot undergo metabolic degradation unless it enters cells. PGE_2_ exists as a monoanion at physiological pH, and it is not permeable to plasma membranes [10,11]. To date, several membrane transporter proteins have been identified that recognize PGs as substrates, and there is compelling evidence indicating that they are involved in regulating the biological actions of PGE_2_ [3]. Among the known transporters, SLCO2A1 (also known as PGT, or OATP2A1) contributes predominantly to the cellular uptake of PGE_2_ and determines local and systemic PGE_2_ levels [4]. SLCO2A1 has a comparable affinity for PGE_2_, PGD_2_, and PGF_2α_ [12], and facilitates their clearance from the site of their actions in cooperation with 15-PGDH [13,14]. Therefore, SLCO2A1-mediated uptake is a crucial step for spatiotemporal control of PG signals, in addition to the expression of their specific functional receptors.

In addition to SLCO2A1, several other proteins have been reported to transport PGs, although their physiological contributions remain elusive. Most belong to subfamilies of the organic anion transporting polypeptide (OATP/SLCO) or polyspecific organic cation/anion transporter (SLC22A) in the SLC superfamily (Table 1). In addition, two members of the ATP-binding cassette subfamily C (ABCC), ABCC2 and ABCC4, are well known as primary active transporters for PGs [15]. The physiological and clinical relevance of these transporters remains largely unclear and warrants future investigation. Because SLCO2A1 and MRP4 have been the most extensively characterized, these two transporters are focused on the following sections.

## 5. The Roles and Dynamics of PGE_2_ During Infections Caused by Pathogens

Infection can cause brain inflammation, which is primarily classified into meningitis, encephalitis, and brain abscess. Meningitis and encephalitis are most frequently triggered by viruses and bacteria, and require immediate hospitalization to prevent brain damage and death. Meningitis is a medical condition in which inflammation primarily affects the pia mater and arachnoid mater (i.e., leptomeninges), two of the three layers of meninges that cover the brain and spinal cord. In encephalitis, the direct invasion of pathogens such as viruses, or an abnormal immune response, triggers inflammation in the brain parenchyma. Elevated PG levels caused by activated immune cells are a hallmark of these diseases and serve as a primary cause of severe pain and fever. PGE_2_ stimulates and sensitizes receptors on sensory nerves, thereby lowering the pain threshold [53,54], and induces fever via the sympathetic nervous system [6]. A common feature of patients with meningitis and encephalitis is that elevated inflammatory cytokines along with PGE_2_ disrupt the function of the BBB, causing fluid from blood vessels to leak into surrounding tissues and resulting in edema. This increases intracranial pressure, which causes severe headaches and vomiting. At the site of inflammation, COX-2 and PG synthases are upregulated to enhance the production of PGs, leading to a marked surge of PG levels in the cerebrospinal fluid (CSF). Specifically, PGE_2_ and PGF_2α_ concentrations in the CSF can reach levels of several thousand pg/mL in patients with meningitis [55,56], while levels in healthy individuals’ range in the tens of pg/mL in the CSF; therefore, PGE_2_ and PGF_2α_ could serve as clinical markers for meningitis. PG levels in the CSF are strongly correlated with proinflammatory interleukins (e.g., IL-1β) and lactate in bacterial meningitis [57], with isoprostane (8-iso-PGF_2α_, a metabolite of PGF_2α_) in particular being linked to neuroinflammation [58]. Therefore, anti-inflammatory steroids such as dexamethasone are often used to treat these patients to suppress inflammation and relieve pain. Waste products diffused into the CSF are cleared into the blood across the brain–cerebrospinal fluid barrier (BCSFB), as are PGs produced in the brain.

**Figure 1:**
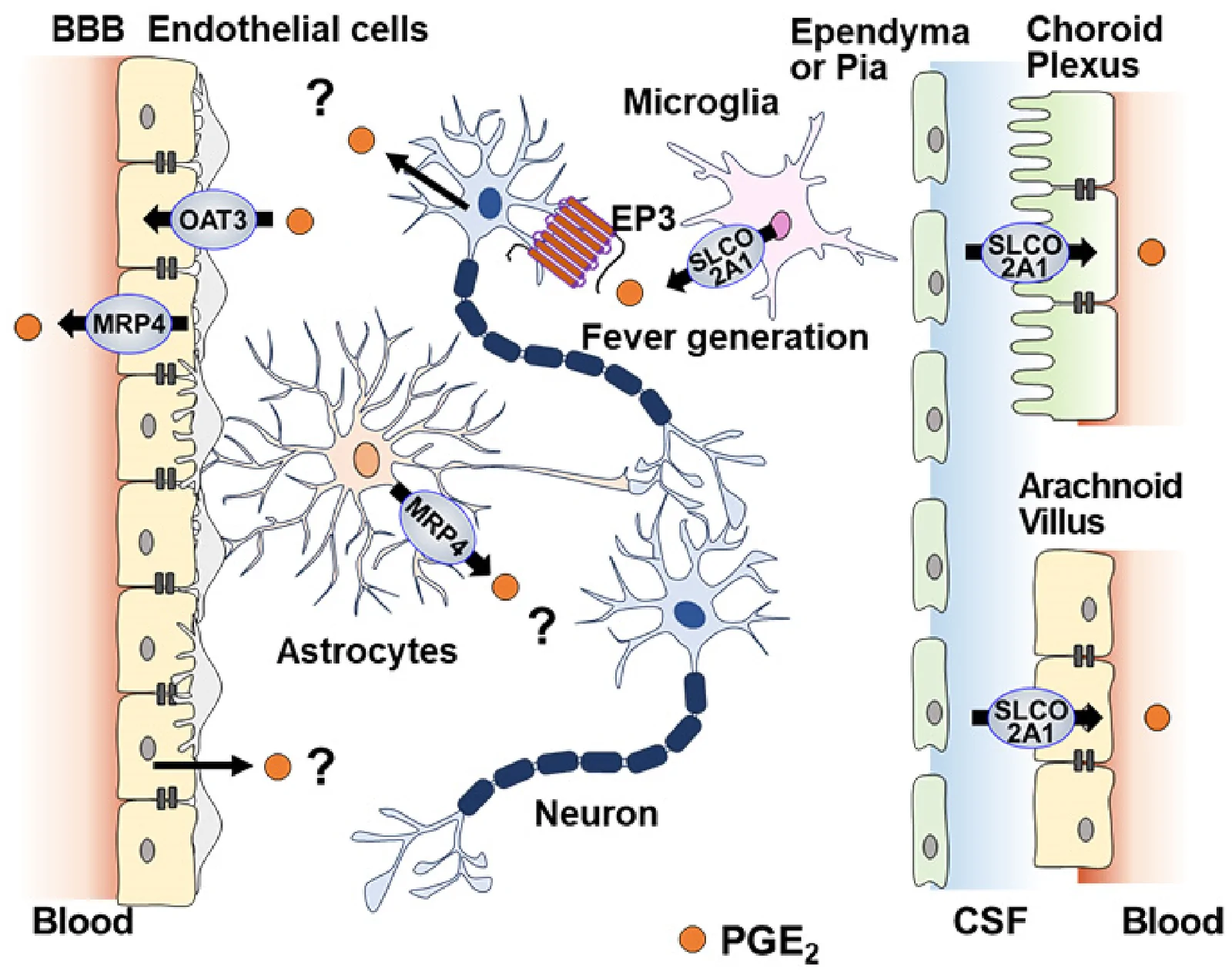
PGE_2_ regulation by membrane transporters. OAT3 and MRP4 are charged to clear PGE_2_ from the brain across the BBB [58]. PGE_2_ (indicated by orange circles) distributed in the CSF could be eliminated by SLCO2A1 expressed in the choroid plexus into the central circulation. SLCO2A1 was shown to be involved in the release of PGE_2_ from the microglia, which could cause a febrile response [64]. Although certain key distribution steps remain to be fully elucidated, the expression of MRP4 in astrocytes has been previously confirmed. However, its specific contribution to PGE_2_ secretion remains elusive.

There are efficient transport systems for PGE_2_ clearance from the brain. MRP4 (ABCC4) has been characterized as an efflux transporter for PGE_2_ in both humans [15] and mice [59]. MRP4 is expressed in brain capillaries in vertebrates [51,60,61,62], and is localized at the luminal membranes of the endothelial cells of brain capillaries in mice [51] and humans [61]. In the abluminal membranes, several PG transporters are involved in the uptake of PGE_2_ from the brain into the endothelial cells. As inhibitors of OAT3 [63] or MRP4 [63] reduce PGE_2_ elimination from the brain, excess PGE_2_ in the brain may be excreted into the blood across the endothelial cells in a unidirectional flow mediated by the MRP4/OAT3 axis (Figure 1). Accordingly, an impaired BBB function may affect this clearance system; however, the dynamics of PGE_2_ may differ when the BBB is compromised under conditions of neuroinflammation (see Section 7.1 and Section 7.3). Another clearance system functions at the BCSFB. Several PG transporters have been shown to be expressed in the choroid plexus epithelial cells of mammals. Studies in rodents have shown that Oat3, Slco1a5, and Slco2a1 are expressed at the apical membranes, while MRP4 is at the basolateral membranes; however, only SLCO2A1 has been shown to be functional for PGE2 clearance. Our laboratory has demonstrated the predominant contribution of SLCO2A1, expressed at the apical membranes, to the clearance of PGE_2_ from the CSF into the blood (Figure 1). Elevated PGE_2_ levels remained in the CSF of *Slco2a1* knockout (KO) mice after i.p. LPS injection, whereas they were quickly reduced to basal levels in wildtype (WT) mice [64]. This observation is consistent with the fact that the elimination rate of radiolabeled PGD_2_, another substrate of SLCO2A1, from the CSF was significantly decreased by inhibiting SLCO2A1 through co-injection of unlabeled PGD_2_ [41,45]. In humans, SLCO2A1 is highly expressed in the choroid plexus according to the Human Protein Atlas project (www.proteinatlas.org; ref. [65]); therefore, SLCO2A1 could be a major determinant of PG concentrations in the CSF of patients. More interestingly, a sophisticated in situ hybridization approach proved that SLCO2A1 is expressed at the apical side of arachnoid membranes, facing the subarachnoidal space, in rats, and is remarkably upregulated at the onset of inflammation [40]; therefore, PG is also expelled into blood vessels from the CSF across the blood–arachnoid barrier (also known as BAB) [66]. Since non-steroidal anti-inflammatory drugs in clinical use inhibit SLCO2A1 [67], attention should be paid to avoiding the inhibition of SLCO2A1-mediated elimination of PGs in infection-associated CNS diseases.

## 6. The Roles and Dynamics of PGE_2_ During Chronic Stress

Chronic stress is the consistent and unceasing response to physiological and psychological stressors, which are classified as external and internal events. External stressors include long-lasting work overload, job insecurity, and financial struggles, whereas internal ones are caused by past trauma and unrelenting anxiety. There is a large body of evidence that repeated environmental stresses harm mental health, inducing anxiety and depression associated with neuroinflammation. The prefrontal cortex (PFC) is a cortical region that is critically involved in higher-order brain functions, such as decision-making, working memory, and emotional control. Rodent models have indicated that the homologous medial PFC (mPFC) is highly susceptible to stress [68,69] and aging [70]. The PFC receives dopaminergic and noradrenergic innervations. Under stress-free conditions, these catecholamines enhance PFC regulation and optimize working memory [71,72], whereas the stress-induced surges in catecholamine impair PFC functions [73,74]. Psychological stress activates the amygdala stress pathways, promoting increased catecholamine secretion from the hypothalamus and brainstem. In humans and rodent models, chronic as well as acute stress also increases the release of known stress hormones, glucocorticoids, from the adrenal gland via activation of the hypothalamic–pituitary–adrenal (HPA) axis [75,76]. Glucocorticoids increase the level of noradrenaline via local glucocorticoid receptors and enhance working memory by reinforcing its action. Conversely, as long-lasting psychiatric stress activates the amygdala, it in turn strengthens fear conditioning and contributes to the reconsolidation of fear memory [77], leading to enhanced glucocorticoid secretion. Elevated levels of glucocorticoids reinforce catecholamine’s action in the PFC by blocking uptake clearance of noradrenaline by organic cation transporter 3 (OCT3, encoded by *SLC22A3*) in the synaptic cleft [78,79]. As a result, normal catecholaminergic signaling supports PFC function, whereas exaggerated catecholamine action in the PFC may weaken neuronal activity and impair working memory, potentially contributing to cognitive and emotional dysfunction implicated in psychiatric disorders, such as attention deficit/hyperactivity disorder, schizophrenia, depression, and PTSD [80,81,82].

**Figure 2:**
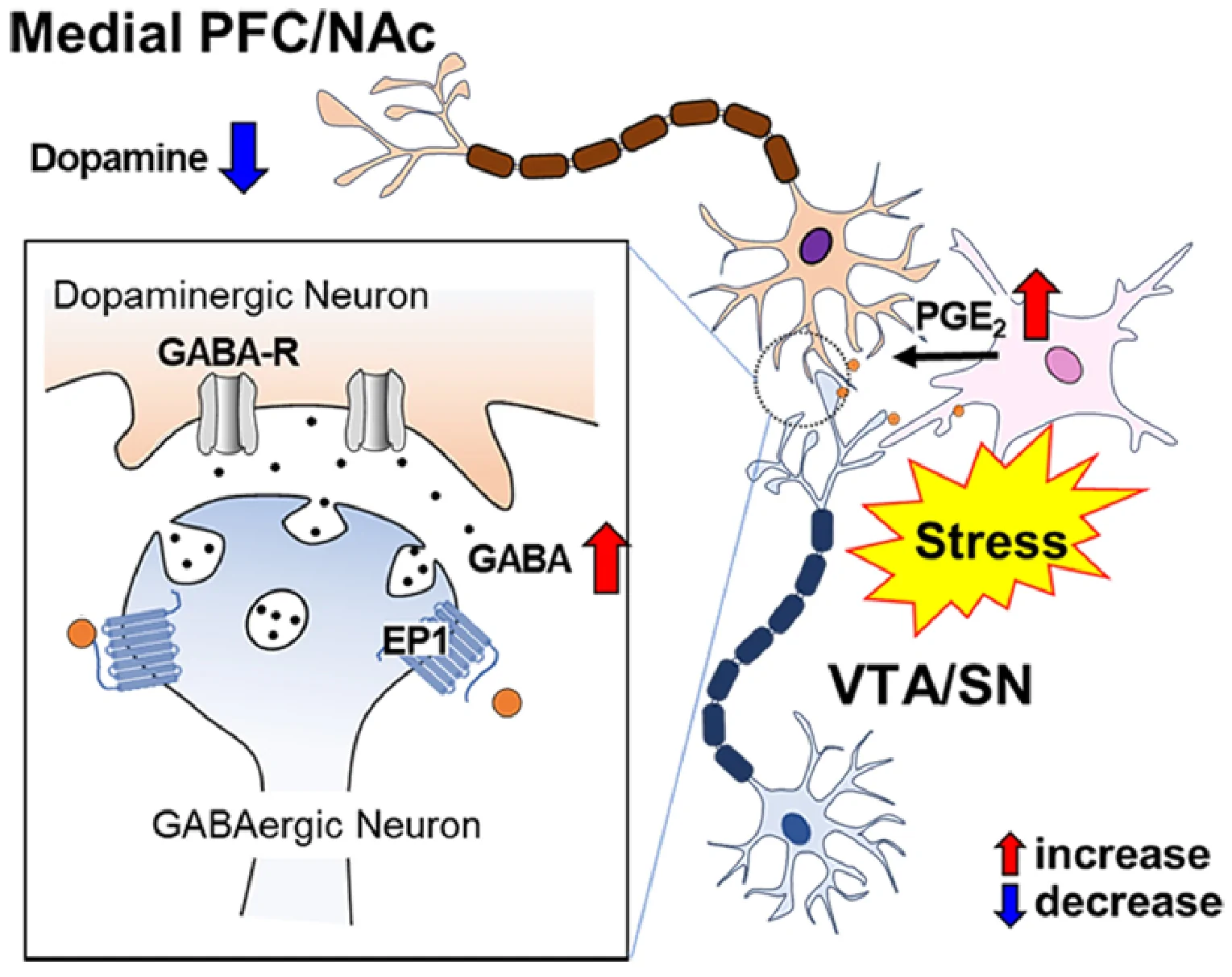
Negative regulation of dopaminergic neurons by PGE_2_. Chronic stress activates microglia via TLR2/4 receptors, causing an enhanced release of PGE_2_ (indicated by an orange circle with red upward arrow) from microglia. Released PGE_2_ stimulates EP1 receptors, which are localized on the pre-synaptic terminals of inhibitory GABAergic neurons. The black arrow indicates the diffusion of PGE_2_ near the synapse. EP1 signaling triggers intracellular Ca^2+^ release, which directly or indirectly promotes the release of GABA (γ-aminobutyric acid, indicated by black dots) from GABAergic nerve terminals. Consequently, GABA accumulates in the synaptic cleft (red upward arrow) and binds to GABA receptors (e.g., GABA-A) located on the cell membranes of dopamine neurons in the VTA and the SN. This enhances GABA-mediated inhibitory input to the dopamine neurons. Consequently, it causes hyperpolarization of the plasma membranes of dopamine neurons and leads to a marked reduction in neuronal excitability, resulting in an inhibition of the release of dopamine from the VTA and SN to projection targets such as the mPFC and the NAc. The blue downward arrow indicates a decrease in dopamine release.

Among the PGs, PGE_2_ plays an especially significant role in the effects of stress on the PFC. Previous research demonstrates that chronic stress stimulates the brain’s immune cells, microglia, via the innate immune receptors TLR2/4, causing them to become active and consistently release PGE_2_ along with inflammatory substances (Figure 2). Microglial activation modifies the dopaminergic innervation in the PFC, eventually causing behavioral changes. Released PGE_2_ suppresses the dopaminergic projection to the mPFC via the PGE receptor EP1. Although the precise molecular mechanism remains undefined, PGE_2_ may mediate an effect via EP1 localized at the GABAergic terminals to augment GABAergic inputs to dopaminergic neurons in the ventral tegmental area (VTA), projecting signals to the mPFC via the mesocortical pathways [83]. Consequently, the cell membranes of dopaminergic neurons undergo hyperpolarization and neuronal excitability is significantly reduced, leading to a decrease in the release of dopamine from the VTA to projection targets such as the medial PFC and the nucleus accumbens (NAc).

In rodent models, the EP1 deficiency causes impulsive aggression and deficits in social behavior in response to psychological stress, demonstrating that PGE_2_-EP1 signaling is crucial for regulating social avoidance behavior associated with psychological stresses [84]. On the other hand, PGE_2_ is closely involved in the secretion of glucocorticoids such as cortisol in humans and corticosterone in rodents. Corticotropin-releasing hormone (CRH)-positive neurons are located in the parvocellular region of the paraventricular hypothalamic nucleus (PVN) and project axons to the median eminence (ME). In response to neuronal activation, the neurons release CRH into the hypophyseal portal system, and CRH induces the release of adrenocorticotropic hormone (ACTH) from the anterior pituitary into the systemic circulation. Intravenous injection of PGE_1_, PGE_2_ or PGF_2α_, but not PGD_2_, increases plasma ACTH levels of in rats [85,86]. Furthermore, the levels are also increased by direct injection of PGE_2_ into several brain sites, including the preoptic anterior hypothalamus [87], the organum vasculosum laminae terminalis [87], and the median eminence [88]. As intracerebroventricular administration of PGE_2_ activates CRH-positive neurons accompanied by an increase in *c-Fos* and *Crh* transcription only in the PVN but not in any other nuclei in rats [89]. Indeed, PGE_2_ injected into the preoptic anterior hypothalamus increases c-FOS expression in PVN as well as the secretion of ACTH [90,91]. These observations suggest that the major potential site of the action for PGE_2_ is the preoptic anterior hypothalamus, but remains undetermined. Thus, PGE_2_ triggers the HPA axis within the CNS, playing an essential role between the immune and nervous systems to modulate neuroendocrine responses.

## 7. The Role of PGE_2_ Signaling in Major Neurological Diseases

**Figure 3:**
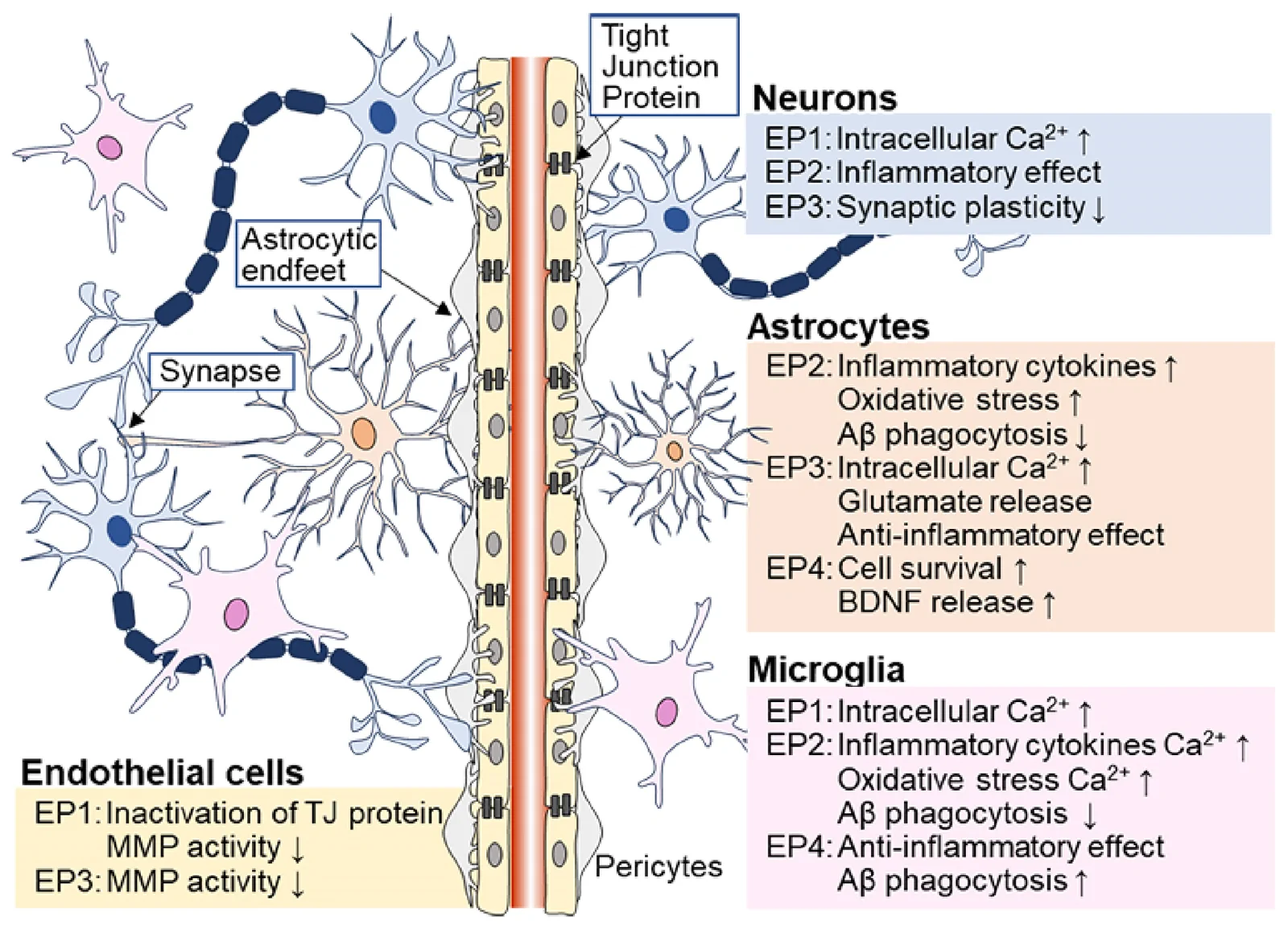
Effects of PGE_2_ on neuroinflammation caused by cerebral stroke and AD. The effects were classified in different cell types. In this Figure, the role of the roles of the respective EP signaling pathways in CNS diseases are summarized for each cell type. Upward and downward-pointing arrows represent an increase or decrease in the indicated effects, respectively.

In the brain, different cell types release PGE_2_ in response to various stressors as an adaptive mechanism. This mediates the so-called sickness response—a suite of symptoms evoked by peripheral inflammation that includes fever, pain, neuroendocrine alterations, and behavioral changes [83]. While blood-borne pro-inflammatory cytokines, which are released from immune cells in response to bacterial infection or immunomodulators, induce COX-2 expression in vascular endothelial cells or perivascular cells [92,93], psychological stress driven by strain, pressure, and anxiety triggers neuronal cells to release PGE_2_ via the COX-2 pathway [94]. In addition to the COX-2 pathway, COX-1 constitutively expressed in microglial cells is considered to be an important source of PGE_2_ by adapting to various psychological stressors [84,95,96]. PGE_2_ release exacerbates neuroinflammation in the brain by amplifying pro-inflammatory mediators and oxidative stress via distinct intracellular signaling pathways coupled to specific receptor subtypes (EP1–EP4). These receptors are heterogeneously expressed on microglia, astrocytes, neurons, and vascular endothelial cells in the brain (Figure 3), and they interact—either cooperatively or independently—to form a chronic inflammatory spiral.

### 7.1. PGE_2_-EP1 Signaling

The EP1 activates phospholipase C and increases the intracellular calcium ion (Ca^2+^) concentration [97]. In neuronal cells, cerebral ischemia induces excitotoxicity through the excessive release of glutamate; this leads to intracellular Ca^2+^ overload and neuronal death. This process is strongly promoted by PGE_2_-EP1 signaling. When PGE_2_ produced by ischemic stroke binds to EP1 expressed in neurons, it acts downstream of *N*-methyl-D-aspartate (NMDA)-type ionotropic glutamate receptors to increase intracellular calcium (Ca^2+^) concentrations [98]. Simultaneously, PGE_2_-EP1 signaling facilitates Ca^2+^ release from intracellular stores. This massive Ca^2+^ overload ultimately triggers neuronal excitotoxicity. Activated microglia produce PGE_2_ through the iNOS-NO pathway, thereby potentially amplifying oxidative stress. The released PGE_2_ subsequently increases intracellular Ca^2+^ levels via EP1 in microglia, causing the Panexin-1 channel to open and release ATP. This amplifies the release of Ca^2+^ from endoplasmic reticulum in microglia [2,99], thereby contributing to inflammatory conditions, although this mechanism can be inhibited by TGF-β1 produced by astrocytes to shut off Panexin-1 [2]. The EP1 antagonist ONO-8713 was shown to improve cell viability in brain slices treated with NMDA [5], which could be attributed to activation of the AKT pathway through PTEN inactivation [100]. Collectively, EP1 is thought to mediate the toxicity associated with induced COX-2 during inflammation, contributing substantially to neurodegenerative and ischemic cell death pathways during stroke. BBB breakdown is a hallmark of ischemic stroke, driven by matrix metalloproteinase (MMP)-mediated degradation of the extracellular matrix tight junction proteins, such as occludin and claudin. Previous studies have shown that the EP1 antagonist SC51089 suppresses the tyrosine phosphorylation of occludin in isolated brain capillaries, thereby protecting the tight junction [101]. Moreover, the blockade of PGE_2_-EP1 signaling was found to significantly reduce MMP-9 and MMP-3 and decrease peripheral neutrophil infiltration in ischemic stroke models [102]. Collectively, the EP1 signaling pathway plays a critical role in BBB breakdown during ischemic stroke.

PGE_2_-EP1 signaling amplifies Aβ-induced neurotoxicity. Zhen et al. demonstrated that EP1-deleted AD transgenic mice exhibited a significant reduction in neuronal damage and memory loss, following ischemic insult [103]. Concomitantly, amyloid plaques burden was alleviated by the deletion of EP1 in these mice, suggesting that EP1 antagonist-mediated prevention of Aβ-associated Ca^2+^ signaling in neurons is neuroprotective. Based on these findings, PGE_2_-EP1 signaling pathway likely contributes substantially to neurotoxicity associated with induced COX-2.

PGE_2_ causes neuropathic pain via EP1. PGE_2_ released at the site of nerve injury or in the spinal dorsal horn acts on EP1 receptors expressed on the central terminals of primary afferent neurons. Elevated levels of PGE_2_ lower the excitation threshold of pain-transmitting neurons, while the sustained release of PGE_2_ may lead to abnormal circuit formation (allodynia), which relays even minor stimuli to the brain as intense pain.

### 7.2. PGE_2_-EP2 Signaling

PGE_2_ produced by inducible COX-2 facilitates the release of proinflammatory cytokines and reactive oxygen species (ROS) by acting on EP2 receptors expressed in astrocytes and microglia. In pathological conditions such as brain inflammation and cerebral ischemia, EP2 expression is rapidly and robustly induced in these glial cells [104] as well as neurons [105]. The results of cell culture experiments have suggested that EP2 receptor activation plays essential roles in activating microglial functions, contributing to neurodegeneration and oxidative stress [106,107,108]. Activated microglia are a major source of a host of proinflammatory and neurotoxic factors including cytokines, chemokines, free radicals, and prostanoids in injured neuronal tissues [1,109]. A previous study showed that, in the middle cerebral artery occlusion–reperfusion model of transient forebrain ischemia, the congenital ablation of EP2 is associated with an increase in the infarcted region in cerebral cortex and subcortical structures, suggesting that PGE_2_-EP2 signaling is neuroprotective in stroke [110]. However, Jiang et al. demonstrated a positive “PGE_2_/EP2/cytokine” loop, in which an increase in COX-2-derived PGE_2_ triggers a rise in the expression of pro-inflammatory cytokines such as IL-1β, IL-6, and TNF-α via the EP2 receptor, which in turn induces further COX-2 upregulation [111]. A recent study revealed that neuronal EP2 also contributes to the progression of cerebral ischemic injury and neurological functions in an ischemic reperfusion mouse model [105]. In astrocytes, PGE_2_-EP2 signaling drives the iNOS-NO pathway via cAMP/PKA/p38 MAPK signaling, suggesting that this crosstalk between inflammatory cytokines and EP2 receptor signaling triggers excessive oxidative stress production [112]. Recently, the pharmacological blockade of EP2 with an antagonist confirmed these reductions, which were associated with improved locomotor and cognitive functions [113]. Collectively, PGE_2_-EP2 signaling amplifies inflammation, and indirectly aggravates neurotoxicity and expands the region of tissue injury in ischemic stroke.

Oxidative damage due to activation of innate immunity in the cerebrum was limited by the deficiency of EP2 in mice [114]. Consistent with this finding, Liang et al. demonstrated amyloid-β (Aβ_40_ and Aβ_42_) deposition remarkably decreased in EP2-deficient AD mouse models, indicating that PGE_2_-EP2 signaling is a key determinant of AD progression [115]. This could be explained by suppressed microglial Aβ clearance due to excessive PGE_2_-EP2 signaling during aging and Aβ accumulation [116]. Concomitantly, it has also been shown that C3 (a complement component) released from astrocytes via EP2 acts on microglial receptors, thereby chronically impairing their Aβ phagocytic capacity. This suggests a key mechanism by which astrocytic inflammatory signaling contributes to the inability of microglia to perform phagocytosis [117]. Furthermore, the aberrant signaling hampers beneficial chemokine production and chemotaxis, nuclear receptor PPARγ signaling, and the IGF1/AKT pathway, ultimately accelerating AD progression [116]. Currently, it is proposed that aging- or AD-associated activation of PGE_2_-EP2 signaling chronically impairs glucose metabolism and mitochondrial homeostasis in macrophages and microglia [118].

PD is classified as a synucleinopathy characterized by the accumulation of misfolded and aggregated α-synuclein—the major component of Lewy bodies [119]—potently activates microglia [120], leading to dopaminergic neurodegeneration through the activation of a membrane-associated nicotinamide adenine dinucleotide phosphate (NADPH) oxidase. An ex vivo experiment has demonstrated that, in EP2 knockout mice treated with a parkinsonian neurotoxin 1-methyl-4-phenyl-1,2,3,6-tetrahydropyridine (MPTP), EP2 ablation significantly enhanced the microglial clearance of α-synuclein aggregates (derived from the mesocortex of Lewy body disease patients), thereby attenuating neurotoxicity and reducing aggregate accumulation [121].

Spinal PGE_2_-EP2 signaling plays a crucial role in amplifying pain by attenuating inhibitory synaptic transmission in the spinal dorsal horn, leading to the exacerbation of neuropathic pain. This role was demonstrated by Reinold et al., who showed that mice deficient in the EP2 receptor did not exhibit the spinal hyperalgesia evoked by PGE_2_ [54]. This is thought to be because glycine receptors, which are responsible for inhibitory glycinergic synaptic transmission, are inhibited via a postsynaptic mechanism driven by EP2 receptor activation [122]. The EP2 receptor on Schwann cells plays a leading role in mediating PGE_2_-driven hyperalgesia in humans and mice. Notably, however, this EP2 pathway is not involved in the inflammatory responses, such as edema and tissue repair, which are essential for tissue protection and recovery. Therefore, targeting EP2 on Schwann cells may be a potential strategy to treating pain without impeding the recovery process [123].

### 7.3. PGE_2_-EP3 Signaling

It has been reported that the EP3 receptor exhibits a dual role: aggravating conditions in cerebral ischemia and AD, while alleviating neuropathic pain. PGE_2_ produced in response to ischemic stress has been shown to exacerbate NMDA-induced neuronal cell death, because EP3 antagonists attenuated it [124], although the underlying mechanism remains unclear. Injury of neuronal cells induced by glutamate receptor agonists, such as kainic acid, upregulates EP3 expression in the endfeet of perivascular astrocytes [125]. PGE_2_, produced by vascular endothelial mPGES induced with a delay after kainic acid treatment, increases intracellular Ca^2+^ levels in astrocytes via the EP3 receptors [126]. This Ca^2+^-dependent glutamate release contributes to the progression of inflammation and neuronal damage in the brain. On the other hand, EP3 receptors expressed in the astrocytes directly mediates a signal that inhibits adenylyl cyclase and reduces the intracellular cAMP levels, thereby counterbalancing the astrocytic inflammatory functions [117]. The spread of inflammation to glial cells and the perivascular region may disrupt the BBB, leading to secondary brain injury and edema. Pharmacological inhibition of EP3 was shown to reduce metalloproteinase-9 [127], which degrades tight junctions and the basement membranes, and genetic deletion of EP3 receptors was shown to ameliorate the damage to the BBB [128]; PGE_2_-EP3 signaling therefore plays a critical role in impairment of the BBB function. Thus, the tight junctions of the BBB could be disrupted via EP1 and EP3 signaling, and the precise clearance system for PGE_2_ across the BBB mediated by MRP4/OAT3 may be affected, thereby altering PGE_2_ dynamics under these conditions, as detailed in Section 3. In AD, PGE_2_-EP3 signaling has been implicated in mediating toxic inflammatory responses. In the mouse AD model, deletion of the EP3 receptor suppressed the induction of proinflammatory gene and protein expression, as well as lipid peroxidation, suggesting a role for the COX-PGE_2_-EP3 signaling axis in AD pathogenesis [129]. PGE_2_ is also related to synaptic plasticity. Long-term potentiation (LTP)—a primary form of synaptic plasticity observed in neural circuits—is considered a cellular basis for learning and memory. This connection is strongly supported by its robust induction in the hippocampus, a central hub for declarative memory. LTP is modulated by intracellular cAMP levels, which are controlled by PGE_2_ signaling via EP2 and EP3 at the neuronal synapses [130]. With aging and Aβ accumulation, PGE_2_ levels increase markedly in regions like the hippocampus. Previously it has been shown that elevated PGE_2_ specifically impairs presynaptic LTP at hippocampal mossy fiber-CA3 synapses via EP3 receptors, by inhibiting adenylate cyclase and consequently reducing intracellular cAMP production [131]. Collectively, the PGE_2_-EP3 signaling pathway impairs synaptic plasticity and cognitive functions.

During inflammation or nerve injury, PGE_2_ induces pain by activating the excitatory receptors EP_2_ and EP_4_, which elevate intracellular cAMP levels. Simultaneously, PGE_2_ stimulates EP_3_ receptors. This EP_3_ activation suppresses cAMP production through a pathway mediated by PI3Kγ, AMPK, and GRK2, serving as an endogenous antinociceptive mechanism that counterbalances the excitatory signals of EP_2_/EP_4_ and reduces the hyperexcitability of primary afferent fibers and spinal dorsal horn neurons [132,133]. However, in chronic nerve injury models, genetic deletion of the EP3 receptor reduced late-stage mechanical allodynia by decreasing the recruitment of immune cells and the subsequent release of CC-chemokine ligand 2. Thus, selective inhibition of EP3 may offer a potential strategy for managing chronic neuropathic pain [134].

### 7.4. PGE_2_-EP4 Signaling

PGE_2_-EP4 signaling pathway has been shown to exhibit a neuroprotective effect in animal models of various CNS disorders disease models such as during cerebral ischemia [135,136], hypoxic–ischemic encephalopathy [137], brain injury [138], and AD [139]. Liang et al. demonstrated that a selective EP4 agonist reduces infarct size and improves neurological deficits [136]. Previous studies have shown that conditional deletion of EP4 in microglia increases lipid peroxidation and proinflammatory gene expression in the brain and in isolated adult microglia treated with lipopolysaccharide (LPS), suggesting that EP4 suppresses inflammatory pathways such as NF-κB. Furthermore, the protective effect of activation of eNOS on cerebral blood flow and blood vessels may contribute to the anti-inflammatory effect of PGE_2_-EP4 signaling [136,137]. Current evidence indicates that PGE_2_-EP4 signaling functions as an endogenous anti-inflammatory mechanism within the brain’s innate immune system (e.g., microglia) by suppressing inflammatory pathways. In astrocytes, the dual nature of PGE_2_-EP4 signaling has been reported during inflammation. Like EP2, it increases cAMP levels; therefore, it functions to promote the secretion of inflammatory factors from astrocytes [140]. At the same time, it may have protective and restorative aspects, such as enhancing the survival of astrocytes themselves via the PI3K/Akt and ERK pathways [141] and partially stimulating the production of neurotrophic factors like brain-derived neurotrophic factor [142]. In addition to these roles in glial cells, EP4 also contributes to the suppression of the spread of inflammation by protecting the BBB from inflammation, since the EP4 agonist L-902,688 has been shown to reduce infarct size and neurological deficits by reducing MMP-3 and MMP-9 [143].

Unlike other EP subtypes, the microglial EP4 receptor has a beneficial effect on the progression of AD in mouse models. In AD, PGE_2_-EP4 signaling suppresses microglial inflammation induced by Aβ at the early stages, thereby facilitating amyloid clearance by microglia. Because EP4 receptor expression is significantly decreased in AD patients, the loss of this anti-inflammatory effect of microglia may contribute to disease progression [139]. In PD, PGE_2_-EP4 signaling likely exhibits neuroprotective effects. Systemic administration of the EP4 agonist ONO-AE1-329 prevented loss of dopaminergic neurons induced by the neurotoxin 1-methyl-4-phenyl-1,2,3,6-tetrahydropyridine (MPTP) in the substantia nigra pars compacta [144]. That report also showed that activation of microglial EP4 suppressed the release of inflammatory cytokines (TNF-α) and chemokines (CCL2 and CCL10) specifically from microglia stimulated by α-synuclein, suggesting a dual neuroprotective and anti-inflammatory action of the EP4 receptor in PD.

Previous studies have described how the EP4 receptor mediates inflammatory pain and sensory neuron excitability induced by PGE_2_ [145,146], and EP antagonists have been shown to suppress inflammatory pain in animal models [147,148,149]. Recently, it was shown that PGE_2_ mediates neuropathic pain by activating adenylate cyclase in injured Aβ dorsal root ganglion (DRG) neurons, where the *I_h_* current, mediated by HCN2 channels, was augmented [150]. Another mouse model study suggests that the PGE_2_-EP4 pathway could activate the TRPV1 channel in DRG neurons, leading to increased neuronal excitability and spinal hypersensitivity [151].

## 8. Transporters Mediated PGE_2_ Transport in the Brain Parenchymal Cells

In the brain, acute and chronic stressors can activate immune cells, leading to neuroinflammation. This response can be mediated by PGE_2_ released from various local cells, facilitating adaptation to conditions including pathogen infection, brain injury and cerebral ischemia, and behavioral changes. Acute stressors, such as peripheral pathogen infection, trigger innate immune cells recruited to the site to release pro-inflammatory cytokines, such as TNF-α and IL-1β, which in turn stimulate the COX-2 pathway in cerebral vascular endothelial and perivascular cells [92]. Chronic stress, generated by feelings of strain and pressure, induces COX-2 in neuronal cells to produce PGE_2_ [94]. While COX-2 is an important mediator of neuroinflammation and CNS diseases, COX-1, which is constitutively expressed in microglia, functions as the primary enzyme for PGE_2_-production under physiological conditions in the brain, particularly in the cerebral cortex and hippocampus, and during early-stage neurotoxicity or stress responses [84,95,96]. PGE_2_ released from these cells is a critical factor in stress adaptation. Several transporters are engaged in PGE_2_ release from cells to regulate its concentration in the vicinity of cell surface EP receptors [15,152]; however, their physiological relevance to CNS diseases remains poorly understood. Of the different transporters studied in the brain, the efflux transporter multidrug resistance-associated protein 4 (MRP4, encoded by *ABCC4*) has been functionally characterized in glial cells. Substantial Mrp4 mRNA expression was detected in astroglia and microglia, whereas less expression was found in neuron and oligodendroglia in the rat brain [153]. MRP4-mediated efflux transport of the substrate drug PMEA (9-(2-phosphonylmethoxyethyl)adenine) was confirmed in the rat MLS-9 microglial cell line [154]. Immunoreactivity for MRP4 was localized to the plasma membrane of BV-2 cells—immortalized mouse microglia [155]—while the expression was significantly reduced in cells exposed to inflammatory stimuli, such as LPS. Thus, MRP4 most likely functions in microglia. Because MRP4 mediates the cellular efflux of both PGE_2_ [15] and cAMP [156], which acts as a second messenger in PGE_2_-EP signaling, the downregulation of MRP4 expression in microglia may contribute to maintaining intracellular levels of cAMP during inflammation, thereby amplifying its signaling cascade. Analyses of post-mortem human brains have reported increased expression of MRP4 protein in regions affected by AD (such as the hippocampus), reflecting gliosis [157]; however, the pathological significance of MRP4 itself is currently under investigation.

Local concentration of PGE_2_ is predominantly regulated by SLCO2A1-mediated cellular uptake of PGE_2_. Choi et al. first reported that SLCO2A1 is expressed in neurons, microglia, and astrocytes in the human brain [25]. A similar distribution was confirmed in the mouse brain [3]. Total protein expression of SLCO2A1 was decreased in the brains of AD patients; however, immunoreactivity for SLCO2A1 appeared more intense within their microglia compared to control subjects [25]. Because SLCO2A1 is a more specific transporter for PGE_2_ than MRP4, its function has been characterized in relation to the cellular distribution of PGE_2_ in macrophages and microglia. SLCO2A1 has been suggested to play a role in the cellular uptake of PGE_2_ in lamina propria macrophages residing in the intestinal tract [158], and an ex vivo experiment using peritoneal macrophages showed its role in the secretion of PGE_2_ out of cells in response to inflammatory stimuli [159]. Our laboratory has shown that Slco2a1 global and macrophage-specific knockout mice exhibit lower levels of PGE_2_ in brain interstitial fluid, suggesting the involvement of SLCO2A1 in PGE_2_ release from activated microglia. However, the underlying mechanism remains poorly understood, warranting future studies to clarify the pathophysiological significance of SLCO2A1 in neurological disorders such as AD.

Other transporters may be involved in PGE_2_ distribution in brain parenchymal cells. Several studies have shown that SLCO family members have distinct, cell-specific patterns within the brain. Human research indicates that SLCO1A2 localizes heavily in frontal cortex and hippocampal neurons, whereas SLCO2B1 appears restricted to brain capillary endothelial cells [18]. Recently, SLCO2B1 was identified as a heme transporter rather than a PGE_2_ carrier, with its expression primarily localized to cerebral microglia [160]. Furthermore, SLCO3A1 variants show regional division: variant v1 stains neuroglial cells in gray matter (sparing neurons), while variant v2 is expressed in gray and white matter neurons (cell bodies and axons) of the human frontal cortex [29]. In that study, the rodent ortholog Slco3a1 was found to be abundant in astroglia. Mouse studies have also revealed the presence of OCT2 in cholinergic neurons, aiding the clearance of neurotransmitters such as noradrenaline and serotonin [161]. OCT2 mediates the transport of diverse endogenous substrates, including PGs, neuropeptides, and steroids, across the plasma membranes of neurons and capillary endothelial cells, implying important roles in cell–cell interactions. PG transporters are crucial determinants of the residence time and concentration of PGE_2_ near its receptor, and so the action of PGE_2_ could be precisely controlled by these membrane proteins. However, much less information is available regarding the roles of these transporters compared to EP receptors. Understanding these roles may provide clues for treating refractory CNS disease.

## 9. Conclusions

In conclusion, PGE_2_ acts as a critical mediator of both acute and chronic brain inflammation, neurodegeneration, and neuropathic pain through its interaction with the EP1–EP4 receptors. While EP1, EP2, and EP3 are well-characterized in neurons, EP2 and EP4 predominantly mediate the response to inflammatory stimuli. Notably, EP1, EP2, and EP3 promote neurodegeneration, whereas EP4 exerts neuroprotective effects in conditions such as ischemic stroke, AD, and PD. Furthermore, PGE_2_ modulates the crosstalk between microglia and astrocytes, steering the progression of neuroinflammation driven by aging and Aβ accumulation in AD. Consequently, subtype-specific EP agonists and antagonists hold significant promises as therapeutic agents for these neurological disorders. Nevertheless, clinical application of PGE_2_ receptor-targeting drugs faces substantial hurdles, particularly regarding BBB permeability and systemic side effects; consequently, current therapeutics remain in the developmental phases.

Current evidence demonstrates that the magnitude and duration of PGE_2_ signaling depend on its local concentration and residence time at the site of action. Indeed, our laboratory recently revealed that pharmacologically blocking SLCO2A1 attenuated the PGE_2_-mediated febrile response, highlighting membrane transporters as viable pharmacological targets. Given the growing consensus that membrane transporters play critical roles in regulating these levels in the brain, elucidating expression profiles of functional PG transporters may provide alternative strategies for modulating PGE_2_ activity, thereby contributing to the treatment of CNS inflammation-based diseases. However, to date, our understanding of the extracellular transporters governing these dynamics remains disproportionately limited compared to our knowledge of their receptors and synthetic enzymes. Future studies should aim to reveal the precise mechanisms governing PGE_2_ influx and efflux, paving the way for novel therapeutic interventions in refractory neurological disorders and neuropathic pains.

## Figures and Tables

**Table 1:** Expression of membrane transporters for PGE_2_ in the CNS.

Family	Species	Gene Symbol	Protein Name	Aliases	Affinity to PGE_2_ (nM)	Ref
SLC	Human	*SLCO1A2*	OATP1A2	OATP-A, SLC21A3	N.D. [16]	[17,18,19],
		*SLCO1C1*	OATP1C1	OATP-F, OATP14, SLC21A14	N.D. [20]	[19,21,22],
		*SLCO2A1*	OATP2A1	PGT, MATR1, SLC21A2, PHOAR2	100 ^b^ [23], 331 ^a^ [24]	[25,26],
		*SLCO2B1*	OATP2B1	OATP-B, SLC21A9	N.D. [20,27]	[18,19,26],
		*SLCO3A1*	OATP3A1	OATP-D, OATP3A1_v1, SLC21A11	56 ^a^ [28], 218 ^a^ [29]	[19,29]
		*SLCO3A1*	OATP3A1_v2	OATP-D, SLC21A11	372 ^a^ [29]	[29]
		*SLCO4A1*	OATP4A1	OATP-E, SLC21A12	N.D. [20,27,30]	[19]
		*SLC22A1*	OCT1	HOCT1	657 ^a^ [31]	[32]
		*SLC22A2*	OCT2	-	28.9 ^a^ [31]	[32,33]
		*SLC22A6*	OAT1	HOAT1, PAHT, ROAT1	970 ^a^ [31]	[34]
		*SLC22A8*	OAT3	-	345 ^a^ [31]	[34]
	Rat	*Slco1a2*	Oatp1a4	Oatp2, Slco1a4, Slc21a5	N.D. [20]	[19,35,36]
		*Slco1a5*	Oatp1a5	Oatp3, Slc21a7	5680 ^a^ [20], 3500 ^a^ [37]	[19,36,38]
		*Slco1b2*	Oatp1b2	Oatp4, Slc21a10	9570 ^a^ [20], 1300 ^a^ [37]	[19]
		*Slco2a1*	Oatp2a1	Matr1, Slc21a2	94 ^a^ [12]	[28,39,40]
		*Slco2b1*	Oatp2b1	Moat1, Slc21a9	N.D. [20]	[35]
		*Slco3a1*	Oatp3a1	OATP-D, Slc21a11	N.D. [28]	[28]
		*Slc22a8*	Oat3	Roct	4240 ^a^ [41]	[35,42,43]
	Mouse	*Slco2a1*	Oatp2a1	Pgt, Slc21a2	110 ^c^ [44]	[45,46]
		*Slc22a8*	Oat3	Roct	1480 ^a^ [47]	[48]
ABC	Human	*ABCC2*	MRP2	CMOAT, cMRP, ABC30, DJS	N.D. [49]	[50]
		*ABCC4*	MRP4	MOAT-B	3400 ^a^ [15]	[15,51]
	Mouse	*Abcc4*	Mrp4	MOATB	N.D. [52]	[15,51]

^a^; Km value, ^b^; K_1/2_ value, ^c^; Ki value. SLC; Solute Carrier, ABC; ATP-biding Cassette Transporter. N.D.; Not determined.

## Data Availability

No new data were created or analyzed in this study. Data sharing is not applicable to this article.

## Abbreviations

EP	Prostanoid E
GPCR	G protein-coupled receptor
PG	Prostaglandin
CSF	Cerebrospinal fluid
BCSFB	Brain cerebrospinal fluid barrier
COX	Cyclooxygenase
PGES	Prostaglandin E synthase
PFC	Prefrontal cortex
mPFC	Medial prefrontal cortex
VTA	Ventral tegmental area
NAc	Nucleus accumbens
SN	Substantia nigra
CRH	Corticotropin-releasing hormone
PVN	Paraventricular hypothalamic nucleus
ACTH	Adrenocorticotropic hormone
NMDA	N-methyl-D-aspartate
MMP	Matrix metalloproteinase
ROS	Reactive oxygen species
NOS	Nitric oxide synthase
Aβ	Amyloid-β
LTP	Long-term potentiation
LPS	Lipopolysaccharide
DRG	Dorsal root ganglion
AD	Alzheimer’s disease
PD	Parkinson’s disease
